# ASSOCIATIONS BETWEEN FUNCTIONAL NETWORKS OF PHYSICAL RESERVE, POSTURAL INSTABILITY, AND WHITE MATTER LESIONS

**DOI:** 10.1093/geroni/igae098.0149

**Published:** 2024-12-31

**Authors:** Chun-Liang Hsu, Roee Holtzer, Roger Tam, Walid Alkeridy, Teresa Liu-Ambrose

**Affiliations:** The Hong Kong Polytechnic University, Hong Kong, Hong Kong, China (People’s Republic); Yeshiva University and Albert Einstein College of Medicine, Bronx, New York, United States; University of British Columbia, Vancouver, British Columbia, Canada; King Saud University, Riyadh, Ar Riyad, Saudi Arabia; University of British Columbia, Vancouver, British Columbia, Canada

## Abstract

White matter hyperintensities (WMH) are clinical markers of subcortical ischemic vascular cognitive impairment (SIVCI) associated with impaired postural balance and falls. Physical reserve (PR) is a recently established construct that reflects one’s capacity to maintain physical function despite brain pathology. This cross-sectional study aims to map functional networks associated with PR, and examining the relationship between PR, WMH, and postural balance. Physical reserve was defined in 22 community-dwelling older adults with SIVCI as the unexplained residual variance in Timed-Up-and-Go test (TUG) after accounting for age, global cognitive function measured by Alzheimer’s Disease Assessment Scale-Cognitive-13 (ADAS-Cog-13), and hippocampal volume. Functional neural networks associated with PR were extrapolated as TUG-correlated network connectivity maps computed using general linear models that removed the effects of age, ADAS-Cog-13, and hippocampus volume. Subsequent analyses examined whether PR and its associated brain networks moderated the relationship between WMH and postural balance under two conditions – eyes open while standing on foam (EOF) and eyes open while standing on floor (EONF). Physical reserve and its associated functional neural networks - frontoparietal network (FPN) and default mode network (DMN) - significantly moderated the association between WMH and postural balance. Specifically, in those with high PR, postural balance was maintained regardless of WMH load while in those with low PR, postural balance worsened as WMH load increased. These results suggest the attenuated effects of WMH on postural stability due to PR may be underpinned by functional neural network reorganization in the FPN and DMN as a part of compensatory processes.

